# Editorial: Immune-Epithelial Crosstalk in Inflammatory Bowel Diseases and Mucosal Wound Healing

**DOI:** 10.3389/fimmu.2018.01171

**Published:** 2018-05-30

**Authors:** Moritz Leppkes, Britta Siegmund, Christoph Becker

**Affiliations:** ^1^Department of Internal Medicine 1, Gastroenterology, Pneumology and Endocrinology, Friedrich-Alexander-University Erlangen-Nürnberg (FAU), Universitätsklinikum Erlangen, Erlangen, Germany; ^2^Department of Gastroenterology, Infectious Diseases and Rheumatology, Charité – Universitätsmedizin Berlin, Berlin, Germany

**Keywords:** inflammatory bowel diseases, intestinal epithelium, immune system, mucosal immunity, lymphocytes

**Editorial on the Research Topic**

**Immune-Epithelial Crosstalk in Inflammatory Bowel Diseases and Mucosal Wound Healing**

## Epithelial Border Patrol

The intestinal surface is covered by a single cell lining of columnar epithelial cells, which are perfectly equipped for tasks in nutrient absorption in the small intestine and water resorption in the colon. As these cells come into contact with a plethora of luminal constituents, the intestinal epithelium also needs to be considered as the gut’s first line of defense under homeostatic conditions. The luminal microflora can be considered as a long neglected additional organ of the body, and alterations in the microbial composition have been implicated as driving elements of multiple intestinal and extraintestinal diseases (1–4). In the wake of this “microbiome era,” it is of utmost importance to elucidate mechanisms, of how immune cells and epithelial cells, on the one hand, react to and, on the other hand, actively shape the intestinal microflora. In this research topic, we introduce the work of several research groups dealing with intestinal immune homeostasis. Epithelial cells are generated from intestinal stem cells at the bottom of the crypts and differentiate into distinct cell types specializing in tasks of either absorption or secretion, respectively: enterocytes are responsible for absorptive functions, whereas goblet cells and enteroendocrine cells fulfill secretory tasks (5). At the bottom of the small intestinal crypt, Paneth cells have been identified by their high granular content as distinct secretory cells, providers of antimicrobial effector molecules and crucial housekeepers of the intestinal stem cell niche (6). The group of Jan Wehkamp and Eduard Stange has substantially contributed to the concept that small intestinal Paneth cells may represent a critical cell type in the pathogenesis of ileal Crohn’s disease. In this research topic, Armbruster et al. explore how monocytes direct the antimicrobial response of Paneth cells by Wnt ligands.

The highly dynamic cellular events of epithelial repopulation along the crypt–villus axis require adaptions of the epithelial cytoskeleton, cell migration, and polarity. GTPases of the Rho family direct actin network remodeling in the intestinal epithelium. Lopez-Posadas et al. have recently published a seminal study, which introduced a role of epithelial prenylation and Rho GTPases to epithelial homeostasis and implied a possible pathogenic role of these processes in inflammatory bowel diseases (IBD) (7). In this research topic, they discuss the regulation of the epithelial cytoskeleton and its adaptive response during inflammatory stress. Patterson and Watson have performed insightful studies on the regulation of intestinal epithelial shedding and its relation to cell death and shed light on this cellular process under homeostatic and inflammatory conditions (8).

The intestinal epithelium represents the first responder to microbial assaults and is thus functionally equipped to detect microbial intruders. Coleman and (Haller) provide a concise overview on how epithelial cells sense microbial components on a molecular level and what we have learned from gnotobiotic mice. One possible consequence of pattern recognition is the assembly of multimeric protein complexes in epithelial cells, known as inflammasomes. Lei-Leston et al. focus on this specific host-protective mechanism of epithelial cells, which has raised a tremendous amount of interest in past years (9).

The epithelial response to inflammatory insults is not only governed by direct effects of pathogenic microorganisms. Khalil et al. discuss the role of transient receptor potential channels in guiding neuropeptide release and immune cell activation in experimental models of colitis (10). Furthermore, tissue-resident mesenchymal cells subjacent to the epithelial barrier fulfill multiple tasks in the cellular crosstalk at mucosal barriers. Here, Kurashima et al. shed light on various mechanisms of how tissue-resident mesenchymal cells instruct epithelia and educate the intestinal immune response.

## Intestinal Immune Cell Populations—Variable Reaction Forces

The human body is equipped with a plethora of humoral and cellular mechanisms on how to resist external hazards. The intestinal tract harbors an enormous quantity and various well-known and yet to be defined immune cell populations, which respond to microbial challenges (11). T cell populations have attracted abundant attention and represent the primary target of successful therapeutic strategies in the treatment of IBD (12). Various strategies have evolved and target activation (azathioprine, cyclosporine, and anti-TNF) and differentiation (anti-IL-12/IL-23) of effector T cells, as well as their homing to the intestinal mucosa (anti-integrins). In this part of the series, Konjar et al. discuss the contribution of intestinal CD8 T cells to intestinal immune homeostasis. Intestinal T cell responses are subject to tight checks and balances. Effector T cell responses are suppressed by regulatory T cell populations, which enforce intestinal immune homeostasis (13, 14). In this issue, Wiesinger et al. provide an update on efforts to restore the balance of effector and regulatory T cells in ulcerative colitis by adoptive transfer of *ex vivo* expanded patient-derived autologous regulatory T cells. Kempski et al. discuss how specific effector cells, CD4^+^ Th17 cells, orchestrate epithelial adaptions to specific inflammatory and neoplastic cues (15). Before being able to give rise to tissue-destructive immune responses, T cells need to home to the mucosa by transendothelial migration (Zundler et al.). Zundler et al. focus on molecular and functional mechanisms of T cell homing to the intestinal mucosa and the effects of anti-integrin strategies (Fuchs et al.).

Apart from understanding disease-driving molecular mechanisms, it is instrumental to discover ways to resolve inflammation (16). Ungaro et al. emphasize the role of specific lipid mediators in this process that actively determine the resolution phase of inflammation.

*A picture is worth a thousand words*. Waldner et al. provide insights into state-of-the-art methods on how to visualize inflammation and immune–epithelial crosstalk both *ex vivo* and *in vivo* in clinical applications using advanced imaging techniques including multiphoton microscopy and endomicroscopy. They describe the current state of the art and novel translational efforts to make the most out of advanced optical tools and their use in predicting the response to therapy.

Taken together, in this research topic, we propose that IBD develop as the consequence of a dysregulated immune–epithelial communication. Insufficient handling of environmental stressors by the intestinal epithelium would thus induce a devastating T-cell-guided immunopathology. The integrated approach of this research topic, linking immunology to epithelial biology, highlights avenues on how to advance the field for the future benefit of affected patients.

## Author Contributions

ML drafted the manuscript. All the authors edited the manuscript.

## Conflict of Interest Statement

The authors declare that the research was conducted in the absence of any commercial or financial relationships that could be construed as a potential conflict of interest.
